# Detection of loss of heterozygosity at RAD51, RAD52, RAD54 and BRCA1 and BRCA2 loci in breast cancer: pathological correlations

**DOI:** 10.1038/sj.bjc.6690722

**Published:** 1999-10

**Authors:** R Gonzalez, J M Silva, G Dominguez, J M Garcia, G Martinez, J Vargas, M Provencio, P España, F Bonilla

**Affiliations:** Departments of Medical Oncology, Pathology, Clinica Puerta de Hierro, Madrid, Spain; Department of Pathology, Hospital Santa Cristina, Madrid, Spain

**Keywords:** RAD51, RAD52, RAD54, BRCA1, BRCA2, LOH

## Abstract

Loss of heterozygosity (LOH) in loci of the 15q15.1, 12p13, 1p32, 17q21 and 13q12–13 regions may collaborate in the inactivation of RAD51, RAD52, RAD54, BRCA1, BRCA2 and possibly other genes implicated in the repair of double-stranded DNA and in DNA recombination. We investigate allelic losses in microsatellites of the RAD51, RAD52, RAD54, BRCA1 and BRCA2 regions, and their correlations with nine pathologic parameters in 127 breast carcinomas. The LOH analysis was performed by amplifying DNA by PCR, using 15 markers of the 15q15.1, 12p13.3, 1p32, 17q21 and 13q12–13 regions. LOH was found in the RAD51 region in 32% of tumours, in the RAD52 region in 16%, in RAD54 in 20% and in the BRCA1 and BRCA2 regions in 49% and 44% respectively. Significant correlations between one or more regions with concomitant LOH and pathologic parameters were observed with respect to age (*P* = 0.008), oestrogen receptor content (*P* = 0.03), progesterone receptors (*P* = 0.003), higher grade (*P* = 0.001), more advanced stage (*P* = 0.004) and peritumoural vessel involvement (*P* < 0.0001). The number of cases in which LOH was observed simultaneously in two or more regions was always higher than expected on the basis of their statistical probability, and curiously, the three patients with LOH at five regions concomitantly were under the age of 30 years. These results suggest that LOH at these regions could be related to breast cancer, and probably to a poor tumour prognosis. © 1999 Cancer Research Campaign


*British Journal of Cancer* (1999) **81**(3), 503–509
© 1999 Cancer Research Campaign
Article no. bjoc.1999.0722

### Detection of loss of heterozygosity at RAD51, RAD52, RAD54 and BRCA1 and BRCA2 loci in breast cancer: pathological correlations

**R Gonzalez[1], JM Silva[1], G Dominguez[1], JM Garcia[1], G Martinez[2], J Vargas[3], M Provencio[1], P España[1] and F Bonilla[1]**

Departments of [1]Medical Oncology and [2]Pathology, Clinica Puerta de Hierro, Madrid, Spain; [3]Department of Pathology, Hospital Santa Cristina, Madrid, Spain

**Summary** Loss of heterozygosity (LOH) in loci of the 15q15.1, 12p13, 1p32, 17q21 and 13q12–13 regions may collaborate in the inactivation of RAD51, RAD52, RAD54, BRCA1, BRCA2 and possibly other genes implicated in the repair of double-stranded DNA and in DNA recombination. We investigate allelic losses in microsatellites of the RAD51, RAD52, RAD54, BRCA1 and BRCA2 regions, and their correlations with nine pathologic parameters in 127 breast carcinomas. The LOH analysis was performed by amplifying DNA by PCR, using 15 markers of the 15q15.1, 12p13.3, 1p32, 17q21 and 13q12–13 regions. LOH was found in the RAD51 region in 32% of tumours, in the RAD52 region in 16%, in RAD54 in 20% and in the BRCA1 and BRCA2 regions in 49% and 44% respectively. Significant correlations between one or more regions with concomitant LOH and pathologic parameters were observed with respect to age ($P$ = 0.008), oestrogen receptor content ($P$ = 0.03), progesterone receptors ($P$ = 0.003), higher grade ($P$ = 0.001), more advanced stage ($P$ = 0.004) and peritumoural vessel involvement ($P$ < 0.0001). The number of cases in which LOH was observed simultaneously in two or more regions was always higher than expected on the basis of their statistical probability and curiously, the three patients with LOH at five regions concomitantly were under the age of 30 years. These results suggest that LOH at these regions could be related to breast cancer and probably to a poor tumour prognosis © 1999 Cancer Research Campaign

**Keywords:** RAD51; RAD52; RAD54; BRCA1; BRCA2; LOH

The repair of double-stranded DNA breaks and recombination are fundamental processes for all living cells. Double-stranded DNA damage may be repaired by homologous recombination with an intact homologue, by the action of recombination and by repair proteins, which are probably present in a similar fashion in various organisms of different evolutionary stages (Park, 1995). Among these proteins, Rad51, Rad52 and Rad54 play a crucial role in human cells, contributing to the aforementioned processes.

Human Rad51 protein is a homologue of the RecA protein of *Escherichia coli*, and its gene maps to chromosome 15q15.1 and encodes a putative 339-amino acid protein. It is transcribed at high levels in thymus, spleen, testis and ovary (Shinohara et al, 1993). Experiments reported by Benson et al (1994) show that human Rad51 protein binds to single and double-stranded DNA and exhibits DNA-dependent ATPase activity to form nucleoprotein filaments, which have been observed under electron microscopy following negative staining.

Human Rad52 protein is essential for DNA double-stranded break repair and meiotic and mitotic recombination, crucial processes for the maintenance of the genetic integrity of living organisms (Park et al, 1996). Studies regarding the function of the Rad52 protein suggest that the Rad52 protein is not required for the initiation of recombination, but is essential for the intermediate stage following the formation of double-strand breaks but before the appearance of stable recombinants (Park et al, 1996). Resistance to ionizing radiation is conferred by the Rad52 protein to monkey cells (Park, 1995). Its gene maps to chromosomal region 12p13.3, and encodes a 418-amino acid protein (Muris et al, 1994).

Human Rad54 protein belongs to a superfamily of DNA helicases that unwind duplex DNA, forming single-stranded DNA, thus making them available for replication, recombination and repair (Mats r -Rogers, 1990). Mutations in genes that encode this protein have been found to be responsible for cance prone syndromes like xeroderma pigmentosum (Sung et al, 1993) and Bloom syndrome (Ellis et al, 1995). Its gene maps to chromosomal region 1p32 and encodes a 747-amino acid protein (Rasio et al, 1997). Northern blot analysis revealed that Rad54 was expressed in testis and thymus, and in low levels in small intestine, colonic mucosa, breast and prostate (Rasio et al, 1997).

The DNA double-strand break repair pathway in mammalian cells in accomplished by multiprotein complexes. That means that specific interaction among these proteins is a reliable fact; thus, specific interaction between Rad51 and Rad52 has been demonstrated, suggesting that Rad52 may modulate the catalytic activities of Rad51 protein, this interaction being species-specific (Shinohara et al, 1993; Shen et al, 1996). Evidence of specific protein–protein association between p53 and human Rad51 protein has been reported (Stürzbecher et al, 1996). More recentl it has been established that BRCA2 and p53 proteins exist in vivo in the same complexes, and that functional interaction between BRCA2 and p53 is a fact, demonstrated by evidence that BRCA2 inhibits p53 transcriptional activity in cancer cells; in this wa

*Received 5 January 1999*
*Revised 17 March 1999*
*Accepted 20 April 1999*

*Correspondence to:* F Bonilla, Molecular Genetics Unit, Clinica del Trabajo, Avd. de la Reina Victoria 21, 28003-Madrid, Spain

**Table 1** Relationship between LOH in the BRCA1, BRCA2, RAD51, RAD52 and RAD54 regions and age and pathologic parameters in breast carcinomas

| Characteristics | | BRCA1 | | | BRCA2 | | | RAD51 | | | RAD52 | | | RAD54 | | |
|---|---|---|---|---|---|---|---|---|---|---|---|---|---|---|---|---|
| | N | I | LOH (%) | *P* | I | LOH (%) | *P* | I | LOH (%) | *P* | I | LOH (%) | *P* | I | LOH (%) | *P* |
| Tumours | 127 | 107 | 53 (49) | | 108 | 47 (44) | | 98 | 31 (32) | | 90 | 14 (16) | | 109 | 22 (20) | |
| Histologic types | | | | | | | | | | | | | | | | |
| IDC | 107 | 96 | 50 (94) | NS | 97 | 42 (89) | NS | 85 | 28 (90) | NS | 75 | 12 (86) | NS | 91 | 19 (86) | NS |
| Miscellaneous | 20 | 11 | 3 (6) | | 11 | 5 (11) | | 13 | 3 (10) | | 15 | 2 (14) | | 18 | 3 (13) | |
| Age | | | | | | | | | | | | | | | | |
| ≤50 | 50 | 39 | 27 (51) | 0.002 | 39 | 23 (49) | 0.01 | 38 | 15 (48) | NS | 37 | 7 (50) | NS | 42 | 12 (55) | NS |
| >50 | 77 | 68 | 26 (49) | | 69 | 24 (51) | | 60 | 16 (52) | | 53 | 7 (50) | | 67 | 10 (45) | |
| Tumour size | | | | | | | | | | | | | | | | |
| ≤3 cm | 94 | 81 | 37 (70) | NS | 87 | 35 (74) | NS | 74 | 21 (68) | NS | 67 | 11 (79) | NS | 82 | 17 (77) | NS |
| >3 cm | 33 | 26 | 16 (30) | | 21 | 12 (26) | | 24 | 10 (32) | | 23 | 3 (21) | | 27 | 5 (23) | |
| Lymph node metastases | | | | | | | | | | | | | | | | |
| ≤3 | 104 | 85 | 34 (64) | 0.0001 | 81 | 24 (51) | <0.0001 | 77 | 24 (77) | NS | 73 | 11 (79) | NS | 88 | 16 (73) | NS |
| >3 | 23 | 22 | 19 (36) | | 27 | 23 (49) | | 21 | 7 (23) | | 17 | 3 (21) | | 21 | 6 (27) | |
| Oestrogen receptors | | | | | | | | | | | | | | | | |
| Positive | 88 | 74 | 30 (57) | 0.005 | 71 | 18 (38) | <0.0001 | 65 | 16 (52) | 0.03 | 61 | 8 (57) | NS | 77 | 13 (59) | NS |
| Negative | 39 | 33 | 23 (43) | | 37 | 29 (62) | | 33 | 15 (48) | | 29 | 6 (43) | | 32 | 9 (41) | |
| Progesterone receptors | | | | | | | | | | | | | | | | |
| Positive | 68 | 57 | 17 (32) | <0.0001 | 58 | 16 (34) | 0.0003 | 48 | 8 (26) | 0.001 | 49 | 4 (29) | 0.03 | 59 | 8 (36) | NS |
| Negative | 59 | 50 | 36 (68) | | 50 | 31 (66) | | 50 | 23 (74) | | 41 | 10 (71) | | 50 | 14 (64) | |
| Histologic grade | | | | | | | | | | | | | | | | |
| I | 27 | 26 | 6 (11) | <0.0001 | 27 | 3 (6) | 0.0004 | 17 | 3 (9) | 0.005 | 16 | 2 (14) | NS | 24 | 0 (0) | 0.02 |
| II | 53 | 42 | 17 (32) | | 41 | 21 (45) | | 41 | 8 (26) | | 38 | 4 (29) | | 40 | 9 (41) | |
| III | 47 | 39 | 30 (57) | | 40 | 23 (49) | | 40 | 20 (65) | | 36 | 8 (57) | | 45 | 13 (59) | |
| Stage | | | | | | | | | | | | | | | | |
| I | 27 | 20 | 4 (8) | 0.01 | 24 | 2 (4) | 0.0003 | 19 | 2 (6) | 0.05 | 17 | 2 (14) | NS | 22 | 0 (0) | 0.02 |
| II | 87 | 76 | 44 (83) | | 78 | 42 (89) | | 70 | 27 (88) | | 64 | 11 (79) | | 76 | 18 (82) | |
| III | 13 | 11 | 5 (9) | | 6 | 3 (7) | | 9 | 2 (6) | | 9 | 1 (7) | | 11 | 4 (18) | |
| Peritumoural vessel involvement | | | | | | | | | | | | | | | | |
| Yes | 61 | 48 | 38 (72) | <0.0001 | 47 | 32 (68) | <0.0001 | 45 | 17 (55) | NS | 46 | 10 (71) | NS | 51 | 13 (59) | NS |
| No | 66 | 59 | 15 (28) | | 61 | 15 (32) | | 53 | 14 (45) | | 44 | 4 (29) | | 58 | 9 (41) | |

Abbreviations: IDC, invasive ductal carcinoma; N, number of tumours studied; I, informative cases for markers of the corresponding region; LOH, loss of heterozygosity.

coexpression of Rad51 enhances BRCA2's inhibitory effects (Marmorstein et al, 1998).

An aspect that has originated a great deal of interest is the interaction between BRCA1/BRCA2 and Rad51 proteins. The functions of BRCA1 and BRCA2 have not been completely defined, but there is evidence to support their involvement in transcriptional regulation (Chapman and Verma, 1996; Milner et al, 1997) and in DNA repair functions (Scully et al, 1997; Sharan et al, 1997). At least in the mouse, they are required for embryonic development (Ludwig et al, 1997; Suzuki et al, 1997), and BRCA1 has also been reported to transactivate the expression of the cyclin-dependent kinase inhibitor $p21^{WAF1/CIP1}$ in a p53-independent manner, inhibiting cell-cycle progression into the S phase (Somasundaram et al, 1997). However, although mutational studies have linked BRCA1 and BRCA2 genes with hereditary breast and ovarian cancer, their aetiopathogenic mechanisms have not been clearly demonstrated in nullizygous mice (Connor et al, 1997; Ludwig et al, 1997; Suzuki et al, 1997).

Interaction between Rad51 and BRCA1 has been demonstrated through co-immunoprecipitate assays showing that the two proteins are located together in the cell nucleus in both ordinary cells and in cells undergoing meiosis (Scully et al, 1997). Conceivably, then, mutations in their genes could lead to cancer by allowing cells to accumulate potentially dangerous mutations. In the same way, Rad51 interacts with BRCA2, and coexpression of the two genes has been shown in mouse embryos (Sharan et al, 1997), suggesting that structural alteration of either gene could alter the integrity of the DNA repair mechanism.

Mutations in Rad51, Rad52 and Rad54 genes in human tumours have not been reported, but a high rate of loss of heterozygosity (LOH) in the chromosomal region of Rad54 (1p32) was observed in breast cancer (Rasio et al, 1997). We postulated that, in sporadic breast cancer, allelic losses in the chromosomal regions of RAD51, RAD52, RAD54, BRCA1 and BRCA2 genes could be notoriously found, as well as, combined losses of several regions in the same tumours. At the same time, the relation of these molecular alterations with clinicopathologic features of the patients has been investigated.

### METHODS

### Tumour samples and DNA extraction

During the period from January 1997 through May 1998, 127 tumours and corresponding normal tissues were snap-frozen in liquid nitrogen immediately after resection. All specimens underwent histological examination by two pathologists to: (a) confirm the diagnosis of adenocarcinoma; (b) confirm the presence of tumour and evaluate the percentage of tumour cells comprising these samples; and (c) carry out the pathological staging. All samples showed at least 75% of tumour cells. To ascertain if contamination with normal cells in our tumour samples may influence

*© 1999 Cancer Research Campaign*

**Table 2** Relationship between breast carcinomas with LOH in one or more of the five regions studied and tumours without LOH at any region with respect to age and pathologic parameters

| Characteristics | With LOH | (%) | Without LOH | (%) | *P* |
|---|---|---|---|---|---|
| Tumours | 70[a] | | 16[b] | | |
| Histologic types | | | | | |
| IDC | 62 | (88.5) | 14 | (87.5) | NS |
| Miscellaneous | 8 | (11.5) | 2 | (12.5) | |
| Age | | | | | |
| ≤50 | 34 | (48.5) | 2 | (12.5) | 0.008 |
| >50 | 36 | (51.5) | 14 | (87.5) | |
| Tumour size | | | | | |
| ≤3 cm | 51 | (73) | 14 | (87.5) | NS |
| >3 cm | 19 | (27) | 2 | (12.5) | |
| Lymph node metastases | | | | | |
| ≤3 | 51 | (73) | 15 | (94) | NS |
| >3 | 19 | (27) | 1 | (6) | |
| Oestrogen receptors | | | | | |
| Positive | 42 | (60) | 14 | (87.5) | 0.03 |
| Negative | 28 | (40) | 2 | (12.5) | |
| Progesterone receptors | | | | | |
| Positive | 28 | (40) | 13 | (81) | 0.003 |
| Negative | 42 | (60) | 3 | (19) | |
| Histologic grade | | | | | |
| I | 9 | (12.9) | 8 | (50) | 0.001 |
| II | 26 | (37.1) | 6 | (37.5) | |
| III | 35 | (50) | 2 | (12.5) | |
| Stage | | | | | |
| I | 7 | (10) | 7 | (44) | 0.004 |
| II | 56 | (80) | 8 | (50) | |
| III | 7 | (10) | 1 | (6) | |
| Peritumoural vessel involvement | | | | | |
| Yes | 43 | (61.4) | 1 | (6) | <0.0001 |
| No | 27 | (38.6) | 15 | (94) | |

Abbreviations: IDC, invasive ductal carcinoma; LOH, loss of heterozygosity. [a]Informative cases for at least one marker of one region and presence of LOH in it. [b]Informative cases for at least one marker in all regions studied and no presence of LOH in any of them.

the allelic signal intensity with respect to the value considered for the presence of LOH, we randomly selected 20 tumour samples among the 127 cases, and microdissection was performed, removing from them, if present, the normal tissue. After that, DNA was extracted from microdissected and non-microdissected paired tumour samples, and an LOH assay was done, to assess the possible correlation between the two sets of results. DNA was extracted from paired normal and tumour samples using a non-organic method (Oncor. Inc., Gaithersburg, MD, USA).

### Pathological parameters analysed

The following parameters were obtained from the medical records of the 127 patients: birth date, tumour size, lymph node metastases, presence of steroid receptors (oestrogen and progesterone), histological type, peritumoural vessel involvement, pathologic stage and histological grade. Tumour size, axillary lymph node metastases and stage were assessed using the TNM classification. All tumours were graded with regard to the Bloom–Richardson grading system that includes tubule growth pattern (high tubular formation, 1; partial tubule growth pattern, 2; little or no tubule formation, 3), nuclear polymorphism (uniform and regular size, 1; moderate pleomorphism, 2; highly pleomorphic with giant nucleus, 3) and mitotic index (<10 mitosis, 1; between 10 and 19 mitosis, 2; and >20, 3). The final grade was determined by adding the two scores: grade 1, 3–5; grade 2, 6–7; grade 3, 8–9. Presence of peritumoural vessel invasion was analysed, and the steroid receptor content (oestrogen and progesterone) was determined by an immunohistochemical procedure, the results of which were considered to be positive when 25% or more of the cells stained positively.

### PCR conditions, primers and LOH assay

Polymerase chain reaction (PCR) was performed in 25-μl volumes using 0.2 units of *Taq* DNA polymerase and 1 × PCR buffer (Promega, Madison, WI, USA), 200 μM dNTP, 30 pmol of each primer and different concentrations of potassium chloride and magnesium chloride depending on the polymorphic marker. A 30-cycle amplification was done in a thermal cycler (Perkin-Elmer, Cetus, Foster City, CA, USA). Three microsatellite markers were used to determine LOH in the 15q15.1 region in tumour and normal tissue, D15S118, D15S214 and D15S1006 (Source: J Weissenbach, Genethon, Whitehead Institute Center for Genome Research). In the RAD52 (12p13.3) region another three polymorphic markers were used to study allelic loss, D12S98, D12S1698 and D12S358 (Source: J Weissenbach, Genethon, Whitehead Institute Center for Genome Research). The LOH study was performed in the 1p32 region using three microsatellite markers, D1S209, D1S411 and D1S207 (Source: J Weissenbach, Genethon,

© 1999 Cancer Research Campaign 

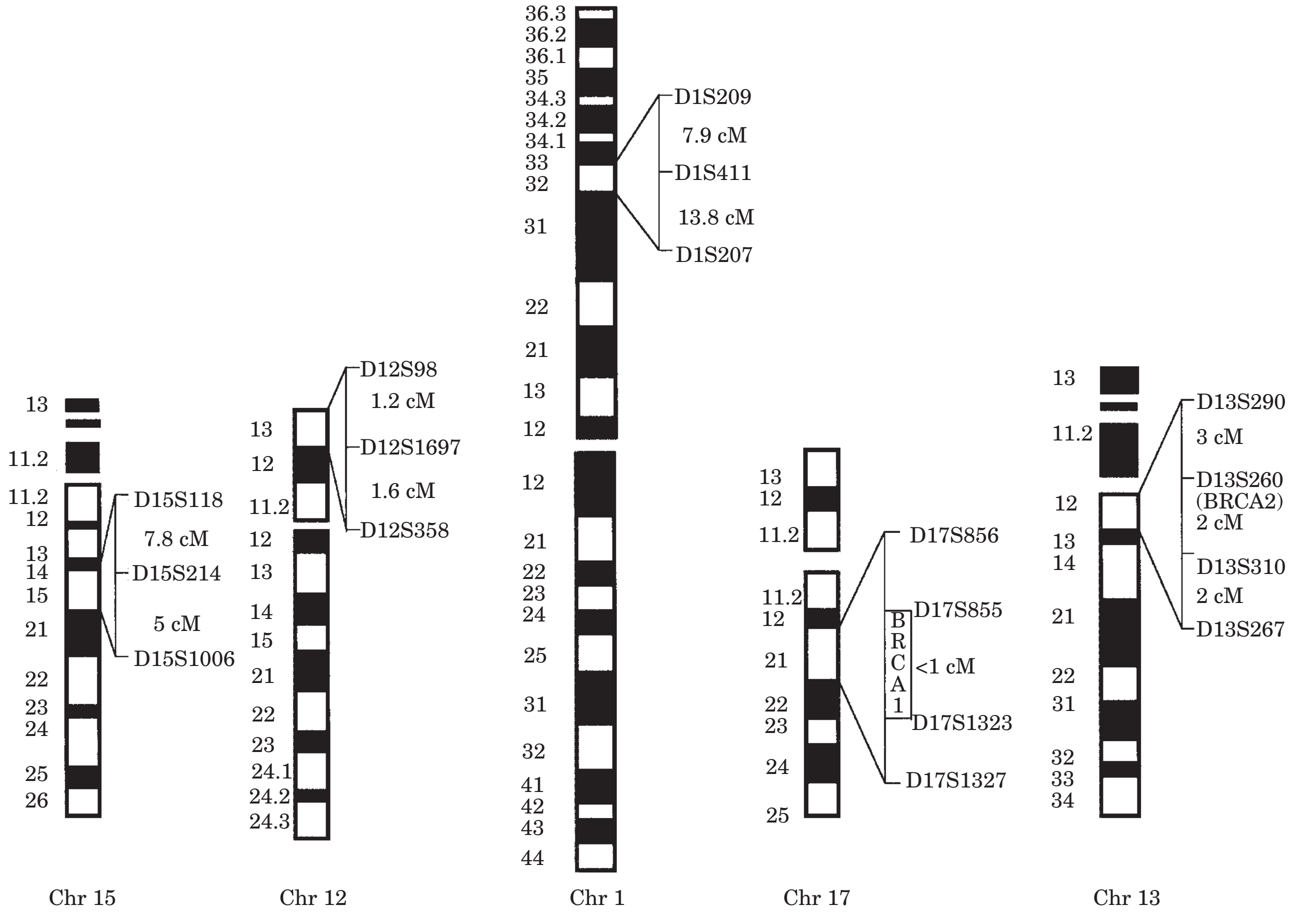

**Figure 1** Illustration of the five chromosomes that harbour the regions studied, as well as, the locations and order of the microsatellite markers used, chosen from the middle or intragenic, if available, and centromeric and telomeric positions in each region. Distances between loci are indicated in centimorgans

Whitehead Institute Center for Genome Research). Four polymorphic markers of the 17q21 region were used, D17S856, D17S855, D17S1323 and D17S1327 (provided by D Goldgar, University of Utah Medical Center, Salt Lake City, UT, USA); another four polymorphic markers were used to study allelic loss in the BRCA2 region, D13S290, D13S260, D13S310 and D13S267 (provided by Fergus J Couch, University of Pennsylvania, Philadelphia, PA, USA), (Figure 1). The annealing temperatures were 55°C for the first nine microsatellites, and 60°C, 58°C, 51°C, 60°C, 55°C, 50°C, 56°C, and 55°C respectively. The alleles were separated by mixing 25 μl of the PCR products with a 10-μl volume of loading buffer (total volume 35 μl), 0.02% xylene cyanol and 0.02% bromophenol blue. Electrophoresis was run on non-denaturing 8–12% polyacrylamide gels for 12–15 h at 500 V. After gel electrophoresis, the allelic band intensity was detected by a non-radioisotopic technique using a commercially available silver staining method (Oto et al, 1993).

### Analysis of LOH

We analysed the allele intensities by densitometry. The gel image was captured using a GS-690 Imaging Densitometer (Bio-Rad Laboratories, Hercules, CA, USA), digitized in 400 dpi, and analysed using Multi-Analyst/PC (Bio-Rad Laboratories, Hercules, CA, USA). An allele was considered to be lost when its signal was reduced by more than 50% with respect to that observed on the normal counterpart DNA (Figure 2). Among the 20 cases studied after microdissection, there were no evaluable decreases in allelic signal intensity to below our cut-off point, 50%. Nearly all the samples maintained a range of signal intensity similar to that exhibited prior to microdissection; there was only one case, that had shown LOH previously, in which the loss of signal intensity changed from 50% to 75%.

### Statistical analysis

The variables analysed were contrasted by means of the $\chi^2$ test with the Yates correction or Fisher's exact test when any of the expected frequencies were less than 5. One-tailed *P*-values of $< 0.05$ were considered statistically significant. Statistical analyses were performed using the EPI-INFO package, version 6.04. The significance, observed for some of the parameters studied in tumours with LOH and tumours without LOH, always related the worst prognosis to tumours with allelic lost. Thus, the incidences of age under 50 years, more than three axillary lymph node metastases, negative steroid receptors, higher histological grade, higher pathologic stage and peritumoural vessel involvement were statistically significant in tumours with LOH.

© 1999 Cancer Research Campaign

### RESULTS

### LOH in the chromosomal regions of the RAD51, RAD52 and RAD54 genes

The 127 eligible breast tumours were analysed for LOH in the regions 15q15.1, 12p13.3 and 1p32, that harbour RAD51, RAD52 and RAD54 genes respectively. Patient age and the pathologic parameters analysed in this study are shown in Table 1. Allelic loss was observed for at least one marker in 31 (32%) of the 98 cases that were informative for microsatellite of the 15q15.1 region; in 14 (16%) of the 90 cases informative for the 12p13.3 region; and 22 (20%) of the 109 cases informative for 1p32 region. When we compared the aforementioned pathological features of the tumours with presence or absence of LOH in the three regions studied, the following parameters showed statistically significant differences: for the RAD51 region, oestrogen receptor content, progesterone receptors, higher grade and higher stage; for the RAD52 region, progesterone receptor content; and for the RAD54 region, higher grade and higher stage. The statistically significant *P*-values for these parameters are shown in Table 1.

### LOH in the 17q21 and 13q12 regions

The same 127 eligible cases were also studied for LOH in the BRCA1 and BRCA2 regions. Patient ages and the pathologic parameters analysed in this study are shown in Table 1. LOH was found for at least one marker in the BRCA1 region in 53 (49%) of 107 cases that were informative for this region. When LOH was studied in the 13q12 region, allelic loss was observed for at least one marker in 47 (44%) of 108 cases that resulted informative for this region. When LOH in these two regions was correlated with age and pathologic findings, we observed statistically significant differences between tumours with and without LOH with respect to the following parameters: age, grouping the patients depending on whether they were older or younger than 50 years, lymph node metastases, oestrogen receptor content, progesterone receptors, higher grade, higher stage and peritumoural vessel involvement. Again, the statistically significant *P*-values for the BRCA1 and BRCA2 regions may be observed in Table 1.

### Pathologic correlations between tumours with LOH and tumours without LOH

When we considered the cases with LOH in one or more of the five regions studied, and compared them with the tumours without LOH in any regions with respect to patient age and histopathological parameters, we found that six parameters were statistically significant: age over or under 50 years ($P = 0.008$), oestrogen receptor content ($P = 0.03$), progesterone receptors ($P = 0.003$), higher grade ($P = 0.001$), higher stage ($P = 0.004$) and peritumoural vessel involvement ($P < 0.0001$); but there was no correlation with regard to histological type, tumour size or lymph node metastases (Table 2). It is noteworthy that the three patients with LOH in all five regions studied were under 30 years of age and showed more than eight affected axillary lymph nodes, negative steroid receptors, histological grade III and presence of peritumoural vessel invasion.

Table 3 Analysis of differences between cases expected with LOH in two or more regions concomitantly and the real cases observed with LOH in the same regions

| Regions studied together | BRCA1 | BRCA2 | RAD51 | RAD52 | RAD54 | F | I | E | O |
|---|---|---|---|---|---|---|---|---|---|
| | + | + | | | | 0.215 | 90 | 19 | 25 |
| | + | | + | | | 0.15 | 84 | 13 | 24 |
| | + | | | + | | 0.076 | 75 | 6 | 10 |
| | + | | | | + | 0.098 | 92 | 9 | 17 |
| 2 | | + | + | | | 0.136 | 80 | 11 | 12 |
| | | + | | + | | 0.067 | 77 | 5 | 6 |
| | | + | | | + | 0.088 | 91 | 8 | 12 |
| | | | + | + | | 0.05 | 74 | 4 | 9 |
| | | | + | | + | 0.063 | 89 | 6 | 11 |
| | | | | + | + | 0.03 | 82 | 2 | 3 |
| | + | + | + | | | 0.068 | 80 | 5 | 9 |
| | + | + | | + | | 0.033 | 66 | 2 | 4 |
| | + | + | | | + | 0.043 | 75 | 3 | 7 |
| | + | | + | + | | 0.023 | 62 | 1 | 7 |
| 3 | + | | + | | + | 0.03 | 71 | 2 | 10 |
| | + | | | + | + | 0.015 | 64 | 1 | 3 |
| | | + | + | + | | 0.021 | 60 | 1 | 6 |
| | | + | + | | + | 0.027 | 82 | 2 | 4 |
| | | + | | + | + | 0.013 | 64 | 1 | 3 |
| | | | + | + | + | 0.01 | 66 | 1 | 3 |
| | + | + | + | + | | 0.01 | 51 | 1 | 3 |
| | + | + | + | | + | 0.013 | 62 | 1 | 4 |
| 4 | + | + | | + | + | 0.0063 | 57 | 0 | 3 |
| | + | | + | + | + | 0.0045 | 49 | 0 | 3 |
| | | + | + | + | + | 0.004 | 51 | 0 | 3 |
| 5 | + | + | + | + | + | 0.002 | 44 | 0 | 3 |

F, probability of LOH at different regions considered simultaneously; I, number of informative cases for the regions studied concomitantly; E, expected LOH cases for each combination of regions; O, real cases observed with LOH in all regions analysed; +, in horizontal, regions examined in unison.

*© 1999 Cancer Research Campaign*

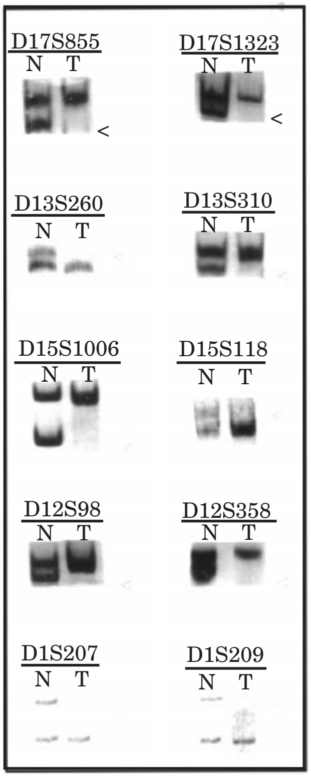

**Figure 2** Photographs of gels, taken under normal light after staining with a $(NO_3)Ag$ method, showing examples of loss of heterozygosity at some of the different loci studied in the 15q15.1, 12p13.3, 1p32, 17q21 and 13q12–13 regions. N, normal tissue; T, tumour; <, allelic loss in tumour DNA

### Analysis of the observed versus expected frequency of concomitant LOH in several regions

We examined the real rate of cases with LOH in two, three, four and five regions simultaneously and compared them with their expected frequency (statistical probability) calculated on the basis of the LOH rate of each region and the number of informative cases for the same region. Briefly, we determined the expected frequency of LOH in BRCA1 and BRCA2 regions together, through the product of the rate of LOH in BRCA1 (49%) and that of LOH in BRCA2 (44%) and by the informative cases for both regions ($n$ = 90), resulting in 19 cases (Table 3). All associations among regions showed more cases observed than expected on the basis of their individual LOH rates; moreover, the proportions between cases expected and observed increased in parallel with the simultaneous regions considered, these being greater if we considered LOH concomitantly in four or five regions rather than in two regions (Table 3).

### DISCUSSION

The functional effect of the loss of one allele of a specific gene, like that analysed in this study, is hypothetical because the loss of the gene function will depend on the inactivation of the other allele by homozygous deletion, promoter methylation or point mutation and, to date, no evidence of somatic RAD51, RAD52, RAD54 or BRCA1 gene mutations has been reported in breast tumours, and they have been detected only exceptionally in the BRCA2 gene (Lancaster et al, 1996; Miki et al, 1996; Teng et al, 1996). However, the identification of chromosomal regions with allelic losses is a useful method for screening genes implicated in the pathogenesis of human malignant tumours, and, at the same time, offers the opportunity to investigate new parameters with possibly high sensitivity and specificity for use as prognostic factors.

In breast cancer, high rates of LOH involving several polymorphic markers in the chromosomal regions of BRCA1, BRCA2 and RAD54 have been detected by other authors (Cleton-Jansen et al, 1995; Beckmann et al, 1996; van der Berg et al, 1996; Rasio et al, 1997) who reported incidences very similar to those observed in this study, but no evidence of LOH in regions of RAD51 and RAD52 genes has been documented. The significant rate of LOH in regions of RAD51 and RAD52 genes here reported, mainly for the RAD51 gene (15q15.1), may indicate that the genes of these regions, not only RAD51 and RAD52, can play a role in the development of breast cancer and in determining the pathologic features of the tumours. Evidence that allelic loss in the BRCA1 and BRCA2 regions is of prognostic value resulted from the comparison of this molecular alteration with pathologic parameters of the tumours (Beckmann et al, 1996; van der Berg et al, 1996), with findings similar to those observed in our series (Table 1). However, no studies are available to contrast our results indicating the significant relation of LOH in RAD51, RAD52 and RAD54 regions with several pathologic parameters, usually accepted as associated with poor prognosis. From the overall point of view, analysing all the cases with LOH in one or more regions concomitantly and the tumours with no allelic loss, the statistically significant differences observed in the individual analysis persisted except in the case of age and lymph node metastases (Table 2). These data suggest that the genes of these regions may influence the specific pathological phenotype of breast carcinomas, either single or combined, because they may perform common or synergistic functions in the control and progression of tumour cells, participating in these processes through the repair of double-stranded DNA and normal chromosomal recombination mechanisms, like those previously reported for BRCA1 and RAD51 (Scully et al, 1997), BRCA2 and RAD51 (Sharan et al, 1997), RAD51 and RAD52 (Shen et al, 1996) or others undetermined.

It is widely accepted that the clinical behaviour of a tumour will be conditioned by its intrinsic biological features (Harris et al, 1997); in this context, LOH of some chromosomal regions, like those here analysed, could also be a molecular factor related to high-grade malignancy, and its determination may help to identify subgroups of patients with a poor prognosis, and eventually to develop more specific treatment strategies.

In breast carcinomas, extensive studies have found many chromosomal regions deleted throughout the genome, suggesting that several putative tumour suppressor genes could be implicated in their pathogenesis, although the number and the relevance of each is currently unknown (Devilee and Cornelisse, 1994; Bièche and Lidereau, 1995; Kerangueven et al, 1997; Phelan et al, 1998). Allelic loss is a genetic alteration that indirectly may indicate the presence of tumour suppressor gene, a phenomenon that apparently can be multiple and originates during tumour progression (Devilee and Cornelisse, 1994; Bièche and Lidereau, 1995). On the basis of this presumption and the aforementioned similar functions of some genes of the chromosomal regions here studied, we analysed whether the rates of concomitant LOH in two or more regions were parallel to its statistical probability, or showed deviations. The cases observed in our series were always higher than expected for the association of the regions considered. The difference expected

© 1999 Cancer Research Campaign and observed between cases increased in a manner that approximated the number of regions with LOH taken together (Table 3). These facts suggest that the presence of two or more regions with deletions in breast carcinomas is not a random event and that it could be related to the development of these malignancies, contributing to their definite pathological phenotype.

### ACKNOWLEDGEMENTS

This work was supported by a grant from the Fundación Central Hispano. We are grateful to David Goldgar and Fergus J Couch for providing the polymorphic markers, to Mrs Martha Messman for her excellent technical assistance and to Mrs Isabel Millán for the statistical study.

### REFERENCES

Beckmann MW, Picard F, An HX, Van Roeyen CRC, Dominik SI, Mosny DS, Schnurch HG, Bender HG and Niederacher D (1996) Clinical impact of detection of loss of heterozygosity of BRCA1 and BRCA2 markers in sporadic breast cancer. *Br J Cancer* **73**: 1220–1226

Benson FE, Stasiak A and West SC (1994) Purification and characterization of the human Rad51 protein, an analogue of *E. coli* RecA. *EMBO J* **13**: 5764–5771

Bièche I and Lidereau R (1995) Genetic alterations in breast cancer. *Genes Chromosomes Cancer* **14**: 227–251

Chapman MS and Verma IM (1996) Transcriptional activation by BRCA1. *Nature* **382**: 678–679

Cleton-Jansen A-M, Collins N, Lakhani SR, Weissenbach J, Devilee P, Cornelisse CJ and Stratton MR (1995) Loss of heterozygosity in sporadic breast tumours at the BRCA2 locus on chromosome 13q12–q13. *Br J Cancer* **72**: 1241–1244

Connor F, Bertwistle D, Mee PJ, Ross GM, Swift S, Grigorieva E, Tybulewicz VLJ and Ashworth A (1997) Tumorigenesis and a DNA repair defect in mice with a truncating Brca2 mutation. *Nature Genet* **17**: 423–430

Devilee P and Cornelisse C (1994) Somatic genetic changes in human breast cancer. *Biochim Biophys Acta* **1198**: 113–130

Ellis NA, Groden J, Ye TZ, Straughen J, Lennon DJ, Ciocci S, Proytcheva M and German J (1995) The Bloom's syndrome gene product is homologous to RecQ helicases. *Cell* **83**: 655–666

Harris JR, Morrow M and Norton L (1997) Malignant tumors of the breast. In: DeVita VT, Hellman S and Rosemberg SA (eds) *Cancer Principles and Practice of Oncology*, Vol. 2, 5th edn, pp. 1557–1616. Lippincott-Noreu, Philadelphia

Kerangueven F, Noguchi T, Coulier F, Allione F, Wargniez V, Simony-Lafontaine J, Longy M, Jackemier J, Sobol H, Eisinger F and Birnbaum D (1997) Genome-wide search for loss of heterozygosity shows extensive genetic diversity of human breast carcinomas. *Cancer Res* **57**: 5469–5474

Lancaster JM, Wooster R, Mangion J, Phelan CM, Cochran C, Gumbs C, Seal S, Barfoof R, Collins N, Bignell G, Patel S, Hamoudi R, Larson C, Wiseman RW, Berchuck A, Iglehart JD, Marks JR, Ashworth A, Stratton MR and Futreal PA (1996) BRCA2 mutations in primary breast and ovarian cancers. *Nature Genet* **13**: 238–240

Ludwig T, Chapman DL, Papaioannou VE and Efstratiadis A (1997) Targeted mutations of breast cancer susceptibility gene homologs in mice: lethal phenotypes of Brcal, Brca2, Brca1/Brca2, Brca1/p53, and Brca2/p53 nullizygous embryos. *Genes Dev* **11**: 1226–1241

Marmorstein LY, Ouchi T and Aaronson SA (1998) The BRCA2 gene product functionally interacts with p53 and RAD51. *Proc Natl Acad Sci USA* **95**: 13869–13874

Matson SW and Kaiser-Rogers KA (1990) DNA helicases. *Annu Rev Biochem* **59**: 289–329

Milner J, Ponder B, Hughes-Davies L, Seltman M and Kouzarides T (1997) Transcriptional activation functions in BRCA2. *Nature* **386**: 772–773

Miki Y, Katagiri T, Kasumi F, Yoshimoto T and Nakamura Y (1996) Mutation analysis in the BRCA2 gene in primary breast cancers. *Nature Genet* **13**: 245–247

Muris DAR, Bezzuvoba O, Buerstedde JM, Vreeken K, Balajee AS, Osgood CJ, Troelstra C, Hoeijmakers JHJ, Osterman K, Schmidt H, Natarajan AT, Eeken JCJ, Lohman PHM and Pastink A (1994) Cloning of human and mouse genes homologous to RAD52, a yeast gene involved in DNA repair and recombination. *Mutation Res* **315**: 295–305

Oto M, Miyake S and Yuasa Y (1993) Optimization of nonradioisotopic single strand conformation polymorphism analysis with a conventional minislab gel electrophoresis apparatus. *Ann Biochem* **213**: 19–22

Park MS (1995) Expression of human RAD52 confers resistance to ionizing radiation in mammalian cells. *J Biol Chem* **270**: 15467–15470

Park MS, Ludwig DL, Stigger E and Lee S-H (1996) Physical interaction between human RAD52 and RPA is required for homologous recombination in mammalian cells. *J Biol Chem* **271**: 18996–18200

Phelan CM, Borg A, Cuny M, Crichton DN, Baldersson T, Andersen TI, Caligo MA, Lidereau R, Lidblom A, Seitz S, Kelsell D, Hamann U, Rio P, Thorlacious S, Papp J, Olah E, Ponder B, Bignon YJ, Scherneck S, Barkardottir R, Borresen-Dale AL, Eyfjörd J, Theillet C, Thompson AM, Devilee P and Larsson C (1998) Consortium study on 1280 breast carcinomas: allelic loss on chromosome 17 targets subregions associated with family history and clinical parameters. *Cancer Res* **58**: 1004–1012

Rasio D, Murakumo Y, Robbins D, Roth T, Silver A, Negrini M, Schmidt C, Burczak J, Fishel R and Croce CM (1997) Characterization of the human homologue of RAD54: A gene located on chromosome 1p32 at a region of high loss of heterozygosity in breast tumors. *Cancer Res* **57**: 2378–2383

Scully R, Chen J, Plug A, Xiao Y, Weaver D, Feunteun J, Ashley T and Livingston DM (1997) Association of BRCA1 with Rad51 in mitotic and meiotic cells. *Cell* **88**: 265–275

Sharan SK, Morimatsu M, Albrecht U, Lim D-S, Regel E, Dinh C, Sands A, Eichele G, Hasty P and Bradley A (1997) Embryonic lethality and radiation hypersensitivity mediated by Rad51 in mice lacking Brca2. *Nature* **386**: 804–810

Shen Z, Cloud KJ, Chen DJ and Park MS (1996) Specific interaction between the human RAD51 and RAD52 proteins. *J Biol Chem* **271**: 148–152

Shinohara A, Ogawa H, Matsuda Y, Ushio N, Ikeo K and Ogawa T (1993) Cloning of human, mouse and fission yeast recombination genes homologous to RAD51 and recA. *Nature Genet* **4**: 239–243

Somasundaram K, Zhang H, Zeng YX, Houvras Y, Peng Y, Zhang H, Wu GS, Licht JD, Weber BL and El-Deiry WS (1997) Arrest of the cell cycle by the tumor-suppressor BRCA1 requires the CDK-inhibitor $p21^{WAF1/CIP1}$. *Nature* **389**: 187–190

Stürzbecher HW, Donzelmann B, Henning W, Knippschild U and Buchhop S (1996) p53 is linked directly to homologous recombination processes via RAD51/RecA protein interaction. *EMBO J* **15**: 1992–2002

Sung P, Bailly V, Weber C, Thompson LH Prakash L and Prakash S (1993) Human xeroderma pigmentosum group D gene encodes a DNA helicase. *Nature* **365**: 852–855

Suzuki A, De La Pompa JL, Hakem R, Elia A, Yoshida R, Mo R, Nishina H, Chuang T, Wakeham A, Itie A, Koo W, Billia P, Ho A, Fukumoto M, Hui CC and Mak TW (1997) Brca2 is required for embryonic cellular proliferation in the mouse. *Genes Dev* **11**: 1242–1252

Teng DH-F, Bogden R, Mitchell J, Baumgard M, Bell R, Berry S, Davis T, Ha PC, Kehrer R, Jammulapati S, Chen Q, Offit K, Skolnick MH, Tavtigian SV, Jhanwar S, Swedlund B, Wong AKC and Kamb A (1996) Low incidence of BRCA2 mutations in breast carcinoma and other cancers. *Nature Genet* **13**: 241–244

van der Berg J, Johannsson O, Hakansson S, Olsson H and Borg A (1996) Allelic loss at chromosome 13q12–q13 is associated with poor prognosis in familial and sporadic breast cancer. *Br J Cancer* **74**: 1615–1619

© 1999 Cancer Research Campaign
